# No evidence for rhythmic sampling in inhibition of return

**DOI:** 10.3758/s13414-023-02745-x

**Published:** 2023-08-23

**Authors:** René Michel, Niko A. Busch

**Affiliations:** 1 Institute of Psychology, University of Muenster, Muenster, Germany; 2 Otto-Creutzfeldt-Center for Cognitive and Behavioral Neuroscience, University of Muenster, Muenster, Germany

**Keywords:** Inhibition of return, Attentional sampling, Theta rhythm, Dense sampling, Exogenous attention

## Abstract

When exogenously cued, attention reflexively reorients towards the cued position. After a brief dwelling time, attention is released and then persistently inhibited from returning to this position for up to three seconds, a phenomenon coined ’inhibition of return’ (IOR). This inhibitory interpretation has shaped our understanding of the spatio-temporal dynamics of the attentional spotlight after an exogenous visual cue for more than three decades. However, a recent theory refines this traditional view and predicts that attention rhythmically alternates between possible target locations at a theta frequency, implying occasional returns of attention to the cued position. Unfortunately, previous IOR studies have only probed performance at a few, temporally wide-spread cue-target onset asynchronies (CTOAs) rendering a comparison of these contradictory predictions impossible. We therefore used a temporally fine-grained adaptation of the Posner paradigm with 25 equally and densely spaced CTOAs, which yielded a robust IOR effect in the reaction time difference between valid and invalidly cued trials. We modelled the time course of this effect across CTOAs as a linear or exponential decay (traditional IOR model), sinusoidal rhythm (rhythmic model) and a combination of both (hybrid model). Model comparison by means of goodness-of-fit indices provided strong evidence in favor of traditional IOR models, and against theta-rhythmic attentional sampling contributing to IOR. This finding was supported by an FFT analysis, which also revealed no significant theta rhythm. We therefore conclude that the spatio-temporal dynamics of attention following an exogenous cue cannot be explained by rhythmic attentional sampling.

**Supplementary Information:**

The online version contains supplementary material available at 10.3758/s13414-023-02745-x.

## Introduction

Posner & Cohen (1984) were first to find that a brief visual cue in the periphery facilitates responses to subsequent targets at the cued position only for a brief period: if the target is presented later than approximately 225 ms after the cue, responses to the very same position are delayed. Since Posner et al. (1985) coined this effect "inhibition of return" (IOR), it has become an intensively investigated phenomenon in the field of cognitive neuroscience (in September 2022, "inhibition of return" on PubMed yielded 319 entries in the last decade and 776 entries in total; for reviews see Berlucchi 2006; Klein 2000; Lupiáñez, Klein and Bartolomeo 2006). While IOR is traditionally explained with a reflexive orienting of attention towards the cued position, followed by a release and persistent avoidance to return to this position (Posner et al., 1985), the recent "rhythmic theory of attention" (Fiebelkorn and Kastner, 2019) refines this pervasive interpretation by predicting at least occasional returns back to the cued position. Therefore, the current study wants to contrast these predictions and test whether IOR can be better explained by a rhythmically reorienting spotlight of attention (VanRullen et al., 2007) rather than by a persistent bias against the previously cued position.

**Fig. 1:**
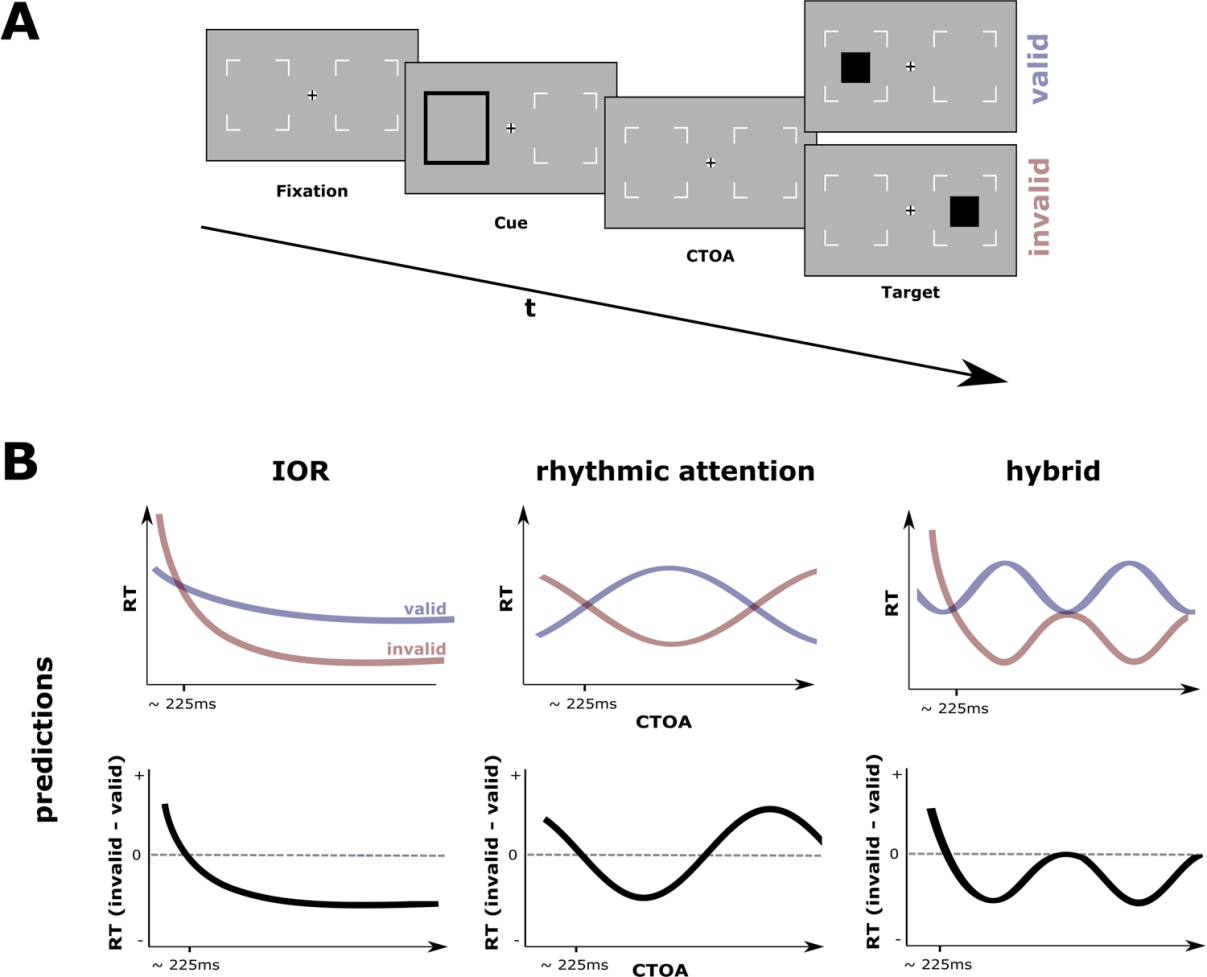
Classic IOR paradigm and results predicted by different theories. (A) Typical exogenous spatial cueing paradigm to investigate IOR (proportions modified for illustration). After a fixation period, a brief cue appears in the periphery at one of the two target positions, i.e. an amplification of the potential target position’s placeholder. After a variable CTOA, the target is presented either at the cued (valid) or uncued position (invalid) with equal probability. Particants are usually asked to detect the target and respond as fast as possible. (B) RT time courses for valid and invalid conditions (top row) and RT differences (invalid-valid; bottom row) predicted by different theories. While all predict a facilitation directly after the cue (up to approximately 225 ms), they diverge in their subsequent predictions: Classic IOR theories predict a persistent facilitation for the uncued condition (left column), while the rhythmic theory of attention predicts alternating phases of facilitation for either the cued or uncued condition (center column). Hybrid models represent a mixture of the "inhibitory tagging" of classic IOR theories and a rhythmic component (right column)

Traditionally, IOR is investigated in an exogenous spatial cueing paradigm with two peripheral target positions horizontally flanking a central fixation marker (see Fig. 1A). Typically, one of the potential target positions is briefly cued with 50% validity (i.e. non-predictive). Following the cue, a target is presented with a variable cue-target onset asynchrony (CTOA) either at the cued or uncued position (note that there are also versions with a central target position or a second cue, see Lupiáñez, Klein and Bartolomeo 2006). Participants are asked to press a button as quickly as possible as soon as they detect the target. For short CTOAs, reaction times (RTs) are faster at the cued than at the uncued position, but for CTOAs longer than approximately 225 ms a reverse pattern with slower RTs for the cued position can be observed, indicating an IOR (see Fig. 1B, left column). While the facilitory effect is comparably short-lived, the inhibitory interval is considered relatively long lasting (up to 3000 ms, see Samuel and Kat, 2003).

This two-stage time course is often interpreted as a product of a novelty seeking mechanism (Posner & Cohen, 1984) or foraging facilitator (Klein, 1988; Klein and MacInnes, 1999) whereby attention automatically orients to the cued position, leading to facilited processing at this position. After a brief dwelling period, attention moves away from the cued position developing a bias against returning to it (Berlucchi, 2006). This "inhibitory tagging" is believed to "encourage the sampling of new information in the visual field" (Klein and MacInnes, 1999). In a paradigm with two locations, this mechanism will unfold as follows: after reflexive orienting towards the cued position, attention will move on to the uncued position, as it is the only relevant position left. But after attention has been deployed to the uncued position, where will attention go next? As the inhibitory interval has been found to persist for up to 3 seconds (Samuel and Kat, 2003) and no other task relevant position is left to explore, attention would need to dwell at the uncued position until the "inhibitory tagging" has faded (as displayed in Fig. 1B, left column).

This view has been refined by findings that visual attention rhythmically samples information in space and time at a theta frequency (3-8 Hz; for an overview see VanRullen, 2016), leading to unvoluntary shifts of attention to uncued positions, even in paradigms eliciting sustained attention at the cued position (Fiebelkorn et al., 2013). These rhythmic shifts of attention have been demonstrated in behavioral data in studies using densely sampled CTOAs for endogenous attention (Senoussi et al., 2019), visual search (Dugué et al., 2015, 2017), and in modified versions of the Posner paradigm with exogenous cues (Landau and Fries, 2012; Michel et al., 2021). These studies found theta- and alpha rhythmic fluctuations in the time course of behavioral accuracy across CTOAs. In line with such findings, Fiebelkorn and Kastner (2019) have proposed their "rhythmic theory of attention" arguing that environmental sampling is organized into two alternating states, i.e. a "sampling phase" and a "shifting phase", and these phases are believed to alternate rhythmically at a theta frequency. While the "sampling phase" is characterized by increased perceptual sensitivity, the "shifting phase" shows decreased perceptual sensitivity and increased probability to shift attention overtly (i.e. saccades) or covertly (i.e. in absence of eye movements). This means that overt or covert attention shifts away from the presently attended location will occur with a certain probability depending on the cue validity (i.e. the lower the cue validity, the higher the probability for reorienting of attention). Fiebelkorn and Kastner (2019) suggested that their proposed "intrinsic bias toward shifting away from the presently attended location" is consistent with IOR in Posner-like cueing paradigms, and speculated that "theta-rhythmic sampling contributes to well-known attentional phenomena, such as inhibition of return", but empirical evidence for this relationship is still pending.

Importantly, their theory predicts that attention may move back to the cued position with a certain probability within each "shifting phase", in contrast to the traditional idea of a stable bias against the cued position in an IOR-like paradigm. More precisely, assuming an internal 4 Hz attentional clocking rhythm, there should be a non-zero chance for an attentional shift back to the cued position occuring approximately every 250 ms. Across CTOAs, this should result in an oscillatory pattern in RTs for both valid and invalid trials, with alternating phases of facilitation for either the valid or invalid position (see Fig. 1B, center column). Such a pattern would indicate that the spotlight of attention moved, at least occasionally, back and forth between the two locations.

Previous studies investigating IOR were not able to demonstrate rhythmic attentional shifts back to the cued location during the inhibitory interval due to their insufficiently dense sampling of CTOAs. As an illustrative example, Posner & Cohen (1984) used six different CTOAs, yielding a sampling frequency for the inhibitory interval of only 5 Hz, thereby restricting observable frequencies to a maximum of 2.5 Hz. Notably, subsequent studies of IOR rarely exceed three different CTOAs, or CTOAs exceeding 225 ms were later merged into a single condition rendering an analysis of rhythmicities impossible (Van der Stoep et al., 2017). Unfortunately, the only study investigating IOR using densely sampled CTOAs did not test the performance time courses for rhythmicities (Li et al., 2020).

Conversely, most studies demonstrating rhythmic sampling in exogenous attention using densely sampled CTOAs were not designed to induce IOR. Most of these studies were designed to test accuracy, not RT, in which IOR is most pronounced (Chen et al., 2017; Landau and Fries, 2012; Michel et al., 2021). Furthermore, most studies have used discrimination tasks instead of the simple detection task introduced by Posner & Cohen (1984). Finally, the dense sampling studies by Song et al. (2014) and Su et al. (2021), who found the typical long-lasting IOR in RTs in a discrimination task, were not intended to investigate IOR. Early CTOAs were presented ten times as often as all other probed CTOAs, thereby biasing temporal expectations towards shorter CTOAs. Moreover, they analyzed IOR and rhythmicities in separate analyses, i.e. they either eliminated high frequencies (> 2 Hz) for their IOR analysis (mimicking a sparser sampling of CTOAs, e.g. as observed in typical IOR studies), or eliminated slow trends (0-2 Hz) for their time-frequency analyses, making it impossible to gain insights into the interplay between these phenomena. But it is precisely this interplay of rhythmic sampling (Fiebelkorn and Kastner, 2019) and inhibitory tagging (Klein, 1988; Klein and MacInnes, 1999; Posner et al., 1985) that needs further investigation to clarify which mechanism can best explain the RT time course of exogenous attention in an exogenous spatial cueing task. We therefore argue that a targeted study combining a simple detection task (e.g. as used by the pioneer study from Posner & Cohen 1984) with a dense sampling approach is needed, together with a unified analysis specifically tailored to contrast both candidate mechanisms.

Therefore, the current study aimed to clarify whether the "rhythmic theory of attention" (Fiebelkorn and Kastner, 2019), classical IOR theories (Klein, 1988; Klein and MacInnes, 1999; Posner et al., 1985) or a combination of both (see Fig. 1B, right column, for an example) can best explain the RT time course of exogenous attention in an exogenous spatial cueing task. To this end, we followed the recommendations from Lupiáñez (2010) for an optimal experimental setup to elicit IOR, but additionally sampled performance with densely spaced CTOAs to be able to capture rhythmic patterns over CTOAs, if present. After fitting common linear or exponential decay models (IOR models), a sinusoidal model (rhythmic model) and a combination of both models (hybrid model) to the RT difference time course (invalid - valid) across CTOAs, we evaluated the models by formal model comparisons (Farrell and Lewandowsky, 2018; Myung et al., 2016). If the linear or exponential decay model turns out to win the comparison, it can be interpreted as evidence in favor of classical IOR theories, whereas a winning sinusoidal model would provide evidence for the rhythmic explanation of IOR. However, if the hybrid model wins the model comparison, it would provide evidence for both phenomena jointly contributing to the time course of reflexive attention in spatial cueing paradigms.

## Methods

The methods and analysis plan have been peer-reviewed and registered prior to data collection and were carried out as planned unless stated otherwise. Technical details can be found in the Supplemental Materials. All code and data are publicly available (see “Availability of data and materials” and “Code availability”).

### Participants

Thirty-nine participants with normal or corrected-to-normal vision (30 female, 33 right-handed, 25 right-eye dominant, aged 18-29 years, $M_{age} = 21.6$, $SD_{age} = 2.4$) were included in the analysis, fulfilling the a-priori determined sample size (see section “Power Analysis” in the Supplemental Materials). Another five participants were tested, but excluded because they exceeded the preregistered exclusion criterion of a 20% false alarm rate. Please note that our number of participants exceeded sample sizes of most dense sampling studies ($\le 16$ participants, e.g. see Benedetto and Morrone 2017; Benedetto et al. 2018; Benedetto and Morrone 2019; Dugué et al. 2015, 2017; Fiebelkorn et al. 2013; Ho et al. 2019; Landau and Fries 2012; Michel et al. 2021; Senoussi et al. 2019; Tomassini et al. 2015).

### Procedure

The study comprised a single recording session of approximately 1 h-duration to collect a total of 660 trials. Prior to the experiment, ten to thirty practice trials to familiarize with the task were presented. Each session was divided into 6 blocks of 110 trials, separated by breaks (self-paced, but at least 30 s), and each block was intermitted by a small break after 55 trials (self-paced, but at least 10 s). A total of 25 CTOAs ranging from 42 ms to 1050 ms in steps of 42 ms were tested with 12 trials per CTOA and validity condition (600 trials in total). Additionally, 60 catch trials (9.1%) without a target were presented, equally distributed across blocks. Cue position, target position and CTOAs were counterbalanced at block level.

The trial sequence and stimulus arrangement is schematically illustrated in Fig. 1A (for details, see “Stimuli” in the Supplemental Materials). Each trial started with a fixation cross and two placeholders for target locations (positioned at 8° eccentricity) for 800 to 1200 ms (randomized across trials), followed by a non-informative visual cue (cue validity: 50%) that was flashed for 100 ms around one of the two target locations. After a variable CTOA, the target was briefly presented for 33 ms either at the cued location (valid) or uncued location (invalid). Placeholders and fixation marker remained on-screen until participant’s response. Participants were asked to press a button as fast and accurately as possible within 2 seconds whenever they detect a target, regardless of target location. Trials with responses prior to target onset were aborted and repeated at the end of the respective block. After response, participants received feedback indicating whether their response was correct. Subsequently, a blank screen was presented as inter-trial-interval for a random duration from 1000 to 1500 ms.

### Fixation monitoring

Participants were required to keep fixating the central fixation marker during the interval from 300 ms after fixation marker onset until target offset. Online fixation monitoring by means of a stationary eye-tracker (see “Apparatus” in Supplemental Materials) automatically aborted trials in which fixation has been broken to repeat them at the end of the respective block. Broken fixations were defined as eye movements > 2° away from the center of the fixation marker or blinks.

## Results

### Preprocessing

**Fig. 2:**
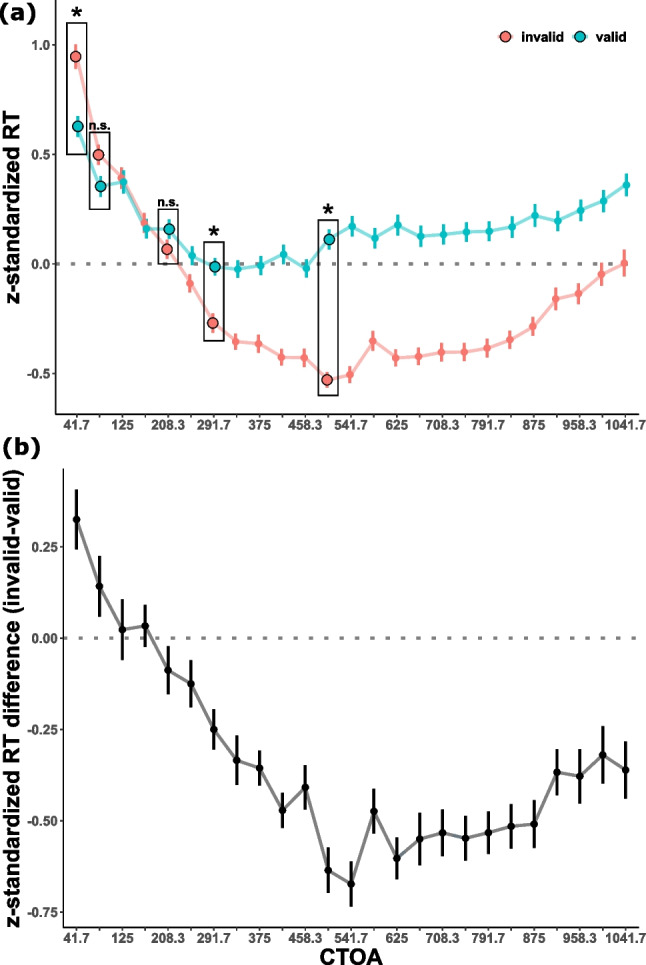
(a) Grand average time course of z-standardized RTs as a function of validity across all probed CTOAs. Error bars indicate standard errors according to Morey (2008). Black boxes highlight the CTOAs that were used for the repeated-measures ANOVA to replicate the original IOR finding by Posner & Cohen (1984). Asterisks above the boxes indicate significant Bonferroni-corrected post-hoc tests, while "n.s." indicates non significant tests. (b) Grand average difference time course (invalid - valid) of z-standardized RTs across CTOAs. Error bars indicate standard errors. The gray dotted line indicates the zero line. Thus, positive values above the line reflect facilitation, whereas negative values below the line indicate inhibition of the valid position

After excluding bad trials (for details, see “Exclusions” in the Supplemental Materials), an average of 11.5 out of 12 trials per participant ($Min = 7$, $Max = 12$) for each of the probed CTOA and validity combinations remained for subsequent analyses. After exlusion, RTs were normalized within participants by z-scaling RT values by subtracting the participant’s mean and dividing by the the participant’s standard deviation, regardless of CTOAs and validity condition. Subsequently, we calculated mean RTs per validity condition, CTOA and participant (see Fig. 2A for the resulting time courses).

### Replicating IOR

To confirm the presence of an IOR effect, we aligned our analysis with the original study by Posner & Cohen (1984). To this end, we approximated their CTOAs (50, 100, 200, 300 and 500 ms, i.e. except the 0 ms) with the best-matching CTOAs in our study (i.e. 42, 84, 210, 292 and 500 ms, highlighted in black boxes in Fig. 2A). The corresponding normalized RTs were subjected to a repeated-measures ANOVA with the within-factors *validity* and *CTOA* (Greenhouse-Geisser corrected; Greenhouse and Geisser, 1959; for details about main effects see Supplemental Materials). Importantly, we found a significant interaction effect of CTOA and validity ($F(3.71, 141.05) = 27.4$, $p < .001$, $\eta ^{2}_{G} = .204$). As predicted, Bonferroni-corrected post-hoc tests showed significantly faster responses in valid trials for the shortest CTOA ($t(38) = 4.01$, $p = .001$), whereas responses were significantly faster in invalid trials for longer CTOAs (292 ms: $t(38) = -3.78$, $p = .003$; 500 ms: $t(38) = -9.92$, $p < .001$). No significant difference was found for the 84 ms ($t(38) = 1.82$, $p = .387$) and 210 ms CTOA ($t(38) = -1.25$, $p > .99$). These results indicate that our paradigm successfully elicited IOR, i.e. the prototypical two-stage time course of facilitation at short CTOAs and inhibition at long CTOAs.

### Model comparison

To contrast the predictions from classic IOR theories and the rhythmic theory of attention, we performed formal model comparisons (for details about model fitting and evaluation see “Model comparison” in the Supplemental Materials).

We first calculated differences in normalized RTs between invalid and valid trials separately for each CTOA (see Fig. 2B) and modelled the resulting differences as a function of time (i.e. CTOAs) as follows: (1) an intercept model (with $b_{0}$ as free parameter) served as baseline model. (2) To describe a short-lived facilitation followed by a persisting inhibition effect (see Fig. 1B, left column), a linear (with $b_{0}$ and $b_{1}$ as free parameters) and an exponential decay model (with $b_{0}$, $N_{0}$, and $\tau$ as free parameters) served as two variants of an IOR model. (3) A sinusoidal model (with $b_{0}$, *a*, *f*, and $\phi$ as free parameters) describing a time course resulting from a rhythmically reorienting attentional spotlight served as the rhythmic model (see Fig. 1B, center column). (4) A linear combination of the exponential decay and sinusoidal model served as a hybrid model, describing an inhibition effect with occasional returns to the cued position (see Fig. 1B, right column).

**Table 1:** Model fits

		Parameter estimates							Goodness of fit	
Model	Formula	$b_{0}$	$b_{1}$	$n_{0}$	$\tau$	a	f	$\phi$	AICc	BIC
intercept model	$rt(t) = b_{0}$	-0.34							6.71	8.60
IOR model (linear)	$rt(t) = b_{0} + b_{1}t$	-0.03	-0.57						-6.21	-3.69
IOR model (exponential)	$rt(t) = b_{0} + N_{0} \times e^{\frac{-t}{\tau }}$	-0.51		1.10	0.18				-34.91	-32.04
rhythmic model	$rt(t) = b_{0} + a \times sin(2 \pi ft + \phi )$	-0.35				0.30	1.00	0.74	-15.23	-12.29
hybrid model	$rt(t) = b_{0} + N_{0} \times e^{\frac{-t}{\tau }} + a \times sin(2 \pi ft + \phi )$	-0.48		0.82	0.18	0.14	1.05	0.72	-57.41	-55.47

*Note* Parameter estimates and goodness of fit indices for all tested models. $b_{0,1}$ = beta weights, $N_{0}$ = initial quantity, $\tau$ = exponential time constant, a = amplitude, f = frequency, $\phi$ = phase

**Fig. 3:**
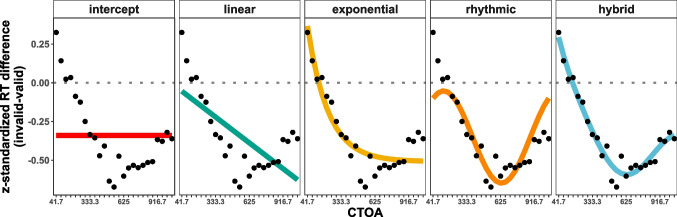
Model fits. Black dots represent the observed data, i.e. the grand average differences of z-standardized RTs (invalid-valid) for each CTOA. Colored lines indicate the predictions of the respective model fit, grey dotted lines indicates the zero line

The hybrid model outperformed all other models (parameter estimates and goodness of fit indices are displayed in Table 1, see also Fig. 3): with regard to the corrected Akaike information criterion (AICc, Akaike 1973; Hurvich and Tsai 1989; Sugiura 1978), we found strong support for the hybrid compared to all other models ($w_{hybrid} > .999$, i.e. the probability of the hybrid being the best model is higher than 99.9%). Importantly the hybrid model was superior to the exponential model ($\Delta AICc = 22.5$) and the rhythmic model ($\Delta AICc = 42.2$). Note that the exponential model also strongly outperformed the rhythmic model ($\Delta AICc = 19.7$). Convergingly, our Bayesian approach also provided decisive evidence for the hybrid compared to all other models ($BF_{hybrid, exponential} > 1000$ , $BF_{hybrid, rhythmic} > 1000$), and also for the exponential compared to the rhythmic model ($BF > 1000$). Correspondingly, at the single participant level, the rhythmic model was amongst the winner models in only 5 out of 39 participants according to AICc (4 according to the Bayesian information criterion, BIC,Schwarz 1978). In contrast, IOR models (linear or exponential) won in 16 cases (14 according to BIC, respectively).

Note that the frequency parameter space of these model fits was restricted to the technically plausible frequency range from 1 Hz to 11 Hz, excluding the Nyquist frequency of 12 Hz. Interestingly, all observed frequency estimates were at the lower bound of this parameter space. To exclude the possibility that the models captured a slow trend and thereby masked a genuinely rhythmic component, we repeated the modelling process and restricted the frequency parameter space to the theta range (3-8 Hz), i.e. the relevant frequency band for our research question^1^. Even then, the exponential model ($w_{exponential} = .981$, i.e. the probability of being the best model is 98.1%) outperformed all other models, with strong evidence compared to both the rhythmic model ($\Delta AICc = 48.89$, $BF > 1000$, model parameters: $b_{0} = -0.35$, $a = 0.08$, $f = 3.32$, $\phi = 6.28$) and the hybrid model ($\Delta AICc = 7.88$, $BF = 32.3$, model parameters: $b_{0} = -0.52$, $N_{0} = 1.13$, $\tau = 0.18$, $a = 0.05$, $f = 3.00$, $\phi = 2.22$). Convergingly, the rhythmic model was not amongst the winner models for any single participant, whereas IOR models (linear or exponential) won in 21 out of 39 cases according to AICc (19 for BIC, respectively).

### Spectral analysis

The model comparison might have disadvantaged small rhythmic effects in the presence of comparably stronger non-rhythmic effects, thereby leading to the incorrect conclusion that rhythmic effects were not present altogether. To test for rhythmicities in RT time courses directly in a way that would not be obscured by stronger non-rhythmic effects, we subjected detrended grand average time courses of the z-standardized RTs across CTOAs to a fast Fourier transform (FFT), separately for valid and invalid condition, as well as the difference time course (invalid-valid; for details about the detrending procedure and permutation tests, see “Spectral Analysis” in the Supplemental Materials).

**Fig. 4:**
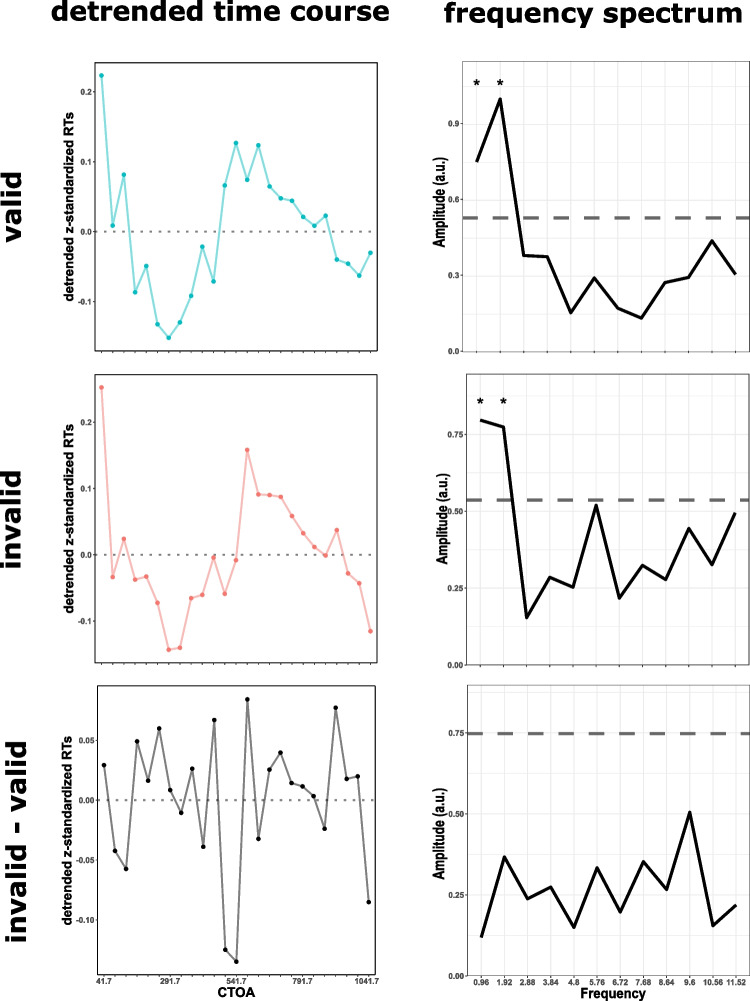
Detrended time courses and spectral analysis results. Left panel: Grand average time courses of z-standardized RTs across all probed CTOAs after second-order detrending. The gray dotted line indicates the zero line. Right panel: Solid black lines represent the amplitude spectrum of the respective detrended grand average time course. Gray dashed horizontal lines indicate the 95th percentile of the respective maximum amplitude distribution (for details see “Spectral Analysis” in the Supplemental Materials, Song et al. 2014; Huang et al. 2015; Huang and Luo 2020; Re et al. 2019). Asterisks indicate significant frequency bins. Top panel: Valid condition. Center: Invalid condition. Bottom: Difference time course (invalid-valid)

The FFT of the valid condition’s detrended grand average time course of the z-standardized RTs revealed significant peaks in the 0.96 Hz and 1.92 Hz frequency bins ($p = .0002$ and $p < .0001$, respectively, see Fig. 4). Likewise, the same frequency bins reached significance for the invalid condition (0.96 Hz: $p = 0.0001$, 1.92 Hz: $p = 0.0001$). Note that the local peak in the 5.72 Hz bin did not reach significance ($p = 0.067$). For the detrended difference time course, none of the frequency bins reached significance.

.

## Discussion

We tested whether inhibition of return (IOR) can be best explained by either the rhythmic theory of attention by Fiebelkorn and Kastner (2019) or by classical IOR theories (Klein, 1988; Klein and MacInnes, 1999; Posner et al., 1985). In a densely sampled exogenous spatial cueing task, we were able to replicate the typical IOR effect with faster RTs in valid compared to invalid trials for short CTOAs (i.e. "facilitation"), and a reverse pattern for longer CTOAs (i.e. "inhibition"). Modelling the time course of this validity effect, model comparisons favored the (exponential) IOR model over a purely rhythmic model. However, a linear combination of an exponential model with a slow 1 Hz component best explained the time course. Contrarily, an FFT analysis of the same time course failed to detect any rhythmicity, although spectra of the separate detrended valid and invalid time courses revealed significant peaks at low frequencies (1-2 Hz).

### Replicating IOR

Our exogenous cueing task clearly elicited the expected IOR pattern (Klein, 2000). Approximating the CTOAs from the original study by Posner & Cohen (1984), we found an initial facilitation effect, i.e. faster RTs for valid trials, followed by an inhibition effect, i.e. the reverse pattern. Traditionally, this is explained by a novelty seeking mechanism (Posner & Cohen, 1984) or foraging facilitator (Klein, 1988; Klein and MacInnes, 1999): attention is initially captured by the cue, leading to an attentional shift towards it and facilitating processing at the cued position. However, after a brief period, attention moves on to the next location. The previously visited location is then tagged for ’inhibition’ to hinder attention’s return to it, thereby encouraging the exploration of new locations in the visual field.

We were also able to confirm the time course of IOR described in the graphical meta analysis pooled over 27 IOR papers by Samuel & Kat (2003, see their Fig. 1) within a single study thanks to our dense sampling approach (see our Fig. 2). While the long-lasting inhibitory phase, starting 292 ms after cue onset, is perfectly in line with the literature (Klein, 2000; Samuel and Kat, 2003), our early facilitation effect seems to vanish a bit earlier than expected: although numerically present, the effect was no longer significant 84 ms after cue onset. In contrast, Posner & Cohen (1984) found facilitation up to 100 ms, and some studies even reported facilitation at CTOAs up to 200 ms (Samuel and Kat, 2003). However, Samuel and Kat (2003) also report that facilitation seems to be stable only in short CTOAs, and many studies fail to show the effect with increasing CTOA duration. Taken together, we were able to replicate the IOR effect and complement the literature with a fine-grained time course of IOR confirming the turning point from facilitation to inhibition around 200-300 ms after cue onset.

### No rhythmic contribution to IOR

In line with the prototypical IOR pattern, the model comparisons clearly favored the non-rhythmic, exponential IOR model over the purely theta-rhythmic model. Even when the frequency parameter space included the delta or alpha frequency range (other studies have found delta and alpha rhythms in comparable experimental setups, e.g., Jia et al. 2017; Song et al. 2014; Su et al. 2021), non-rhythmic IOR models clearly outperformed purely rhythmic models. Likewise, the spectral analysis of the difference time course (invalid-valid) also revealed no significant rhythmic pattern. While the rhythmic theory of attention (Fiebelkorn and Kastner, 2019) would have predicted that the spotlight of attention periodically alternates between target positions, leading to occasional returns of attention back to the initially cued position roughly every 250 ms (see Fig. 1B, left column), our results strongly suggest that a return of the attentional spotlight to the previously cued location is persistently inhibited ($\ge 1~s$, see Fig. 1B, center column). Thus, our results strongly suggest that the time course of IOR can be better explained by a sustained inhibitory mechanism (Klein, 1988; Klein and MacInnes, 1999; Posner et al., 1985) than by theta-rhythmic attentional sampling (Fiebelkorn and Kastner, 2019).

We also tested whether sustained inhibition (Klein, 1988; Klein and MacInnes, 1999) and rhythmic sampling (Fiebelkorn and Kastner, 2019) concurrently contribute to the time course of IOR (see Fig. 1B, right column) by fitting a combination of an exponential IOR model and a rhythmic model. Interestingly, this hybrid model best explained the IOR time course. While this model’s exponential components resemble the exponential IOR model, the rhythmic component revealed a slow frequency of 1 Hz. Although an FFT analysis of the same time course could not confirm the existence of this rhythmicity, both valid and invalid condition separately also showed significant peaks in the 1-2 Hz frequency bins. However, to make the claim that these frequencies constitute a rhythmic pattern, it would have been necessary to observe such a periodicity for multiple cycles, which was not possible in the one-second long time window sampled in this study. Therefore, such low-frequency components might have actually captured aperiodic, but non-rhythmic, trends in the data. For example, looking at Fig. 3, one could speculate that the IOR time course could also be described by a parabola, with the first CTOAs showing a facilitation effect, the middle CTOAs in the vertex reflecting the inhibitory phase, and the upward trend in the last CTOAs potentially foreshadowing the end of the inhibition effect. This trend was perfectly captured by the hybrid model (blue line) almost describing a parabolic trend (within the sampled time window). This could also explain why the FFT analysis of the same time course could not find any rhythmic activity, because the preceding second-order detrending is by design able to remove such a parabolic trend. In addition to such methodological limitations, the 1 Hz component also lacks a theoretical correspondence in the rhythmic theory of attention (Fiebelkorn and Kastner, 2019). Nonetheless, a longer sampling window is needed to better understand the nature of the 1 Hz component, e.g. spanning the 2-3 s inhibitory window suggested by Samuel and Kat (2003). Taken together, we argue that these slow frequencies need to be interpreted with caution.

Importantly, none of our FFT analyses or model fits provided evidence for theta-rhythmic sampling contributing to IOR as speculated by Fiebelkorn and Kastner (2019). Following the seminal studies by Posner & Cohen (1984), we assessed IOR as the speed of behavioral responses to salient stimuli. However, many previous studies have assessed rhythmic sampling as accuracy of responses to low contrast, or near threshold stimuli (e.g. Landau and Fries 2012). Accordingly, several authors have argued that RT are not ideally suited for assessing behavioral oscillations (VanRullen and Dubois, 2011; Kienitz et al., 2022). However, Song et al. (2014) and Su et al. (2021) also used RT as dependent variable in exogenous cueing tasks and were indeed able to find rhythmicities in the alpha range. Furthermore, as IOR is typically measured in RTs, we believe that it was necessary to use them as dependent variable to find out whether rhythmic sampling explains IOR. Moreover, finding no rhythmic pattern in RTs while at the same time clearly observing a long-lasting IOR indeed indicates that the effect of inhibitory tagging affects the IOR time course more than rhythmic sampling, if present at all, by several orders of magnitude. Unfortunately, we were not able to perform separate analysis for left and right-cued trials (e.g. as in Chen et al. 2017; Landau and Fries 2012; Song et al. 2014). While such a hemisphere-specific analysis might in principle increase the sensitivity for behavioral oscillations, it would have critically reduced the signal-to-noise ratio with just six trials remaining per CTOA and hemifield. We conclude that the converging results of our model fits and spectral analyses underline the robustness of the finding that the time course of IOR can be best explained by traditional, non-rhythmic IOR models without any theta-rhythmic contribution.

While our study does not support the speculation that the rhythmic theory of attention (Fiebelkorn and Kastner, 2019) contributes to the phenomenon of IOR, we would like to emphasize that it cannot be interpreted as evidence against this theory in general. While the theta-rhythmic contribution to inhibition following exogenous cues is merely a corollary of the rhythmic theory of attention, the bulk of this theory is concerned with the theta rhythm temporarilly organizing perceptual sampling in alternating phases of increased and decreased perceptual sensitivity. In fact, numerous studies (for an overview see Keitel et al. 2022; Kienitz et al. 2022) have provided support for this central component of the rhythmic theory of attention (Fiebelkorn and Kastner, 2019) and like-minded theories (VanRullen et al., 2007; VanRullen, 2016).

The field currently debates the definition of IOR (Dukewich and Klein, 2015). Specifically, IOR might be a multi-facetted phenomenon with inhibition occurring at the input (i.e., perceptual, attentional) or output (i.e., response) stage, depending on the task (Taylor and Klein, 2000; Redden et al., 2021). We argue that our paradigm most likely elicited the input-based IOR effect by asking for manual responses and prohibiting saccades. Moreover, in similar paradigms, Handy et al. (1999) demonstrated IOR in visual sensitivity, and McDonald et al. (2009) provided electrophysiological evidence for attentional orienting contributing to IOR. Consequently, we argue that in our paradigm inhibition was predominantly due to attentional (re-)orienting, and that our comparison between rhythmic attentional models and attentional IOR models (Posner & Cohen, 1984; Klein, 1988; Klein and MacInnes, 1999) was valid.

### Conclusion

All in all, none of our analyses supported the hypothesis that theta-rhythmic attentional sampling explains or contributes to inhibition of return (Fiebelkorn and Kastner, 2019). Contrarily, we found striking evidence in favor of a two-stage attentional process with an initial attentional shift towards the cued location, followed by a sustained, non-rhythmic inhibition of this location. We therefore conclude that IOR is best explained by an inhibitory mechanism as proposed in traditional models such as the foreaging facilitator (Klein, 1988; Klein and MacInnes, 1999) or novelty seeking mechanism (Posner et al., 1985).

### Supplementary Information

Below is the link to the electronic supplementary material.

Supplementary file 1 (pdf 228 KB)

## Data Availability

Data are available at https://osf.io/b5q2u/.

## Footnotes

1 Note that multiple model fitting procedures were not planned a priori.

## Code Availability

Analysis code is available at https://github.com/remichel/DIOR_analysis/.
