# Supplementary material for: No evidence for rhythmic sampling in inhibition of return
Source: Atten Percept Psychophys. 2023 Aug 23;85(6):2111–21. doi: 10.3758/s13414-023-02745-x (PMC10545570; doi:10.3758/s13414-023-02745-x)
Supplement: Supplementary file 1 — (pdf 228 KB) [file 13414_2023_2745_MOESM1_ESM.pdf]

No evidence for rhythmic sampling in inhibition of return: Supplemental Materials

René Michel[1,2] and Niko A. Busch[1,2]

[1]Institute of Psychology, University of Muenster, Muenster, Germany

[2]Otto-Creutzfeldt-Center for Cognitive and Behavioral Neuroscience, University of Muenster, Germany

No evidence for rhythmic sampling in inhibition of return: Supplemental Materials

Methods

Power Analysis

The Power Analysis to determine the sample size was performed using G*Power (Version 3.1.9.2; Faul, Erdfelder, Buchner, & Lang, 2009). We based our power analysis on the effect size for the *Cue validity x CTOA* interaction ($\eta_p^2 = .096$) from a recent IOR study (Study 2 from Li et al., 2020) and halved it for the subsequent analysis to control for overestimation of reported effect sizes typically found in studies without preregistration (Pavlov et al., 2021; Schäfer & Schwarz, 2019). As required by G*Power, we converted η_p^2 to the effect size

$$f = \sqrt{\frac{\eta_p^2}{1 - \eta_p^2}} \tag{1}$$

and corrected it for repeated measures by multiplying with $\sqrt{2}$ resulting in $f_{corr} = 0.3176$ (Rasch, Friese, Hofmann, & Naumann, 2014). The required sample size to find such an effect size for the planned IOR analysis with a Power $\geq .95$ amounts to 39 participants [1].

Apparatus

Participants performed the experiment in a dimmed room, seated in a fixed chair in front of a calibrated 24" Viewpixx/EEG LCD Monitor (120 Hz refresh rate, 1 ms pixel response time, 95% luminance uniformity, 1920*1080 pixels resolution; www.vpixx.com). A chin rest was used to stabilize the head position and keep the distance to the screen at approximately 86 cm. A stationary eye-tracker (EyeLink 1000+; www.sr-research.com) was used for binocular tracking of the participant's eyes at 500 Hz sampling rate. Calibration of the eye-tracker was carried out using the default nine-point calibration grid. Calibration took place at the beginning of each session and, if necessary, in experiment breaks or when participants had broken fixation in three consecutive trials. Responses were given with a Logitech F310 gamepad (www.logi.com). The experiment was presented using Matlab 2018b (www.mathworks.com) and the Psychophysics Toolbox (Brainard, 1997) on a Linux system

[1] G*Power input to reproduce the results: $\alpha = .05$, $1 - \beta = .95$, $\epsilon = 1$, $N_{measurements} = 5$, $N_{groups} = 1$, correlation among repeated measures = 0; following recommendations from Rasch et al. (2014).

(Intel Core i5–3330 CPU, a 2 GB Nvidia GeForce GTX 760 GPU, and 8 GB RAM). Correct stimulus presentation timing was ascertained by means of a photodiode test prior to the experiment, following recommendations outlined in De Clercq, Crombez, Buysse, and Roeyers (2003).

Stimuli

For an overview of the stimulus arrangement see Figure 1A in the Main Article. All stimuli were presented on a medium gray background (52.2 cd/m^2). Two placeholders indicating target locations (thin square outlines, size = 3° visual angle, linewidth = 0.04°, 102.3 cd/m^2) were positioned at 8° to the left and right of the central fixation marker (diameter = 1°, black and white, 0.2 cd/m^2 and 102.3 cd/m^2; see Thaler, Schütz, Goodale, & Gegenfurtner, 2013). The cue was an amplification of one of the two target location placeholders, i.e. thicker and darker (16.6 cd/m^2, linewidth = 0.08°). The target was a dark square (diameter = 0.1°, 23.7 cd/m^2) centered in one of the two target locations. After the target, only the placeholders and the fixation cross were presented until response. A small colored rectangle (diameter = 0.2°) presented in the center of the screen served as feedback at the end of a trial (yellow for correct, blue for incorrect responses).

Results

The analysis was performed using R (Version 4.1.3) and RStudio (Version 2022.02.1). Data wrangling was carried out using tidyverse packages (Wickham et al., 2019) and the Rmisc package (Hope, 2014). For the analysis of the classical IOR effect, the afex (Singmann, Bolker, Westfall, Aust, & Ben-Shachar, 2022) and emmeans package (Lenth, Singmann, Love, Buerkner, & Herve, 2018) were utilized. For the modelling analysis, nls.multstart (Padfield & Matheson, 2018), broom (Robinson, Hayes, & Couch, 2022) and AICcmodavg (Mazerolle, 2020) were used. For the spectral analysis, custom code was utilized. All scripts are publicly available (see "Code availability" in the Main Article).

Exclusions

At the participant level, we exluded participants, who were not able to complete the recording session, or with a false-alarm rate higher than 20%. Excluded participants were

substituted until we reached the determined sample size (see Section "Participants" in the Main Article).

At the single trial level, we only considered trials with correct responses. Furthermore, we excluded trials that were missed to be rejected by the online fixation control ($Min_{participant} = 0$ trials, $Max_{participant} = 19$ trials, $Mean_{participant} = 2.7$ trials, $SD = 3.7$)[2]. Additionally, we excluded trials with RTs faster than 200 ms. Subsequently, we also excluded trials with RTs exceeding $M \pm 4\ SD$ (separately for each participant and condition, following the procedure by Su, Wang, Kang, & Zhou, 2021). On average, these criteria led to 9.2 additionally excluded trials per subject (SD = 4.3, Min = 4, Max = 19). All in all, trial exclusions led to an average data loss of 3.75% ($SD = 3.3\%$, $Min = 1.2\%$, $Max = 18.3\%$). Importantly, on average 11.5 out of 12 trials ($Min = 7$, $Max = 12$) for each of the probed CTOA and validity combination per participant remained intact for subsequent analyses.

Replicating IOR

Additionally to the significant interaction, we found a significant main effect for validity ($F(1, 38) = 8.42$, $p = .006$, $\eta^2_G = .023$), with faster reaction times for invalid ($M = 346\ ms$, $SD = 38\ ms$) compared to valid trials ($M = 370\ ms$, SD = 41 ms, note that the ANOVA was performed on z-standardized RTs), and a significant main effect of CTOA ($F(3.18, 120.91) = 128.04$, $p < .001$, $\eta^2_G = .563$). Pairwise Bonferroni-corrected post-hoc tests among the selected CTOAs revealed faster responses for longer CTOAs compared to each preceding one (all $t(38) \geq 6.37$ and all $p < .001$), except for the last CTOA (292 ms vs 500 ms: $t(38) = 1.23$, $p > .99$).

Model comparison

For the intercept and linear models, we used the *lm* function from the *stats* package to fit the respective models. For all other models, we used the *nls_multstart* function from the *nls.multstart* package (Padfield & Matheson, 2018) to perform non-linear least squares regression by means of a gridstart approach. To this end, a grid of equally spaced starting values for each of the parameters was tested, and the best fit was determined using AIC

[2] Note that these exclusions have not been preregistered but were in line with the abortion criteria that were a priori defined for the online fixation control, see "Fixation monitoring" in the Main Article.

scores. For each model, this best fit was subsequently used for the formal model comparison between our candidate models.

We evaluated the model fits by means of the Bayesian information criterion (BIC, Schwarz, 1978) and quantified the strength of evidence in favor of the winner model by approximating Bayes factors (BF) on the basis of BIC differences between the candidate models (Wagenmakers, 2007), which can be interpreted according to the guidelines proposed by Raftery (1995). However, due to the BIC's conservative nature and strong punishment for additional parameters, we complemented our BF approach by additionally comparing the models by means of the corrected Akaike information criterion (AICc, Akaike, 1973; Hurvich & Tsai, 1989; Sugiura, 1978). To this end, we quantified the strength of evidence in favor of the winner model by means of AICc differences between the candidate models computing Akaike weights (Wagenmakers & Farrell, 2004) and using the guidelines for interpretation from Burnham and Anderson (2002). Note that the evaluation of more than a single information criterion has been repeatedly recommended (Farrell & Lewandowsky, 2018; Myung, Cavagnaro, & Pitt, 2016) and can provide additional information: while their convergence would be indicative of a moderate or large effect in the data, their divergence would inform about a small or absent effect (as shown in both simulations and empirical data, see Evans, 2019).

Spectral Analysis

The model comparison might have disadvantaged small rhythmic effects in the presence of comparably stronger non-rhythmic effects, thereby leading to the incorrect conclusion that rhythmic effects were not present altogether. To test for rhythmicities in RT time courses directly in a way that would not be obscured by stronger non-rhythmic effects, all time courses were detrended using a second order polynomial (Balestrieri, Ronconi, & Melcher, 2021; Fiebelkorn, Saalmann, & Kastner, 2013; Ho, Leung, Burr, Alais, & Morrone, 2017; Q. Huang & Luo, 2020; Re, Inbar, Richter, & Landau, 2019).

For each time course, we performed a permutation test by generating a distribution of spectral amplitudes under the null hypothesis that there is no temporal structure. To this end, a total of 10,000 permuted datasets[3] were created by shuffling the CTOA labels in the

[3] Note that the Stage 1 Report accidentally stated 100,000 permutations.

respective original dataset at the single trial level within participants (and validity conditions, respectively). The same detrending and FFT as for the original datasets was then carried out for each permuted dataset. Subsequently, to control for multiple comparisons (Nichols & Holmes, 2002), only the maximum amplitude value across frequencies was extracted from each permuted dataset's FFT spectrum to form the distribution under the null hypothesis (Q. Huang & Luo, 2020; Y. Huang, Chen, & Luo, 2015; Re et al., 2019; Song, Meng, Lin, Zhou, & Luo, 2014). We then compared the observed amplitudes against the 95th percentile of this maximum amplitude distribution.

References

Akaike, H. (1973). Information theory and an extension of the maximum likelihood principle. In B. Petrov & F. Caski (Eds.), (pp. 267–281). Budapest: Akademiai Kiado.

Balestrieri, E., Ronconi, L., & Melcher, D. (2021). Shared resources between visual attention and visual working memory are allocated through rhythmic sampling. *European Journal of Neuroscience*. doi:10.1111/ejn.15264

Brainard, D. H. (1997). The psychophysics toolbox. *Spatial vision*, *10*(4), 433–436. doi:10.1163/156856897X00357

Burnham, K. P., & Anderson, D. R. (2002). *Model selection and multimodel inference: A practical information-theoretic approach*. New York: Springer.

De Clercq, A., Crombez, G., Buysse, A., & Roeyers, H. (2003). A simple and sensitive method to measure timing accuracy. *Behavior Research Methods, Instruments, & Computers*, *35*(1), 109–115. doi:10.3758/BF03195502

Evans, N. J. (2019). Assessing the practical differences between model selection methods in inferences about choice response time tasks. *Psychonomic Bulletin and Review*, *26*(4), 1070–1098. doi:10.3758/s13423-018-01563-9

Farrell, S., & Lewandowsky, S. (2018). *Computational modeling of cognition and behavior*. Cambridge University Press.

Faul, F., Erdfelder, E., Buchner, A., & Lang, A. G. (2009). Statistical power analyses using g*power 3.1: Tests for correlation and regression analyses. *Behavior Research Methods*, *41*(4), 1149–1160. doi:10.3758/BRM.41.4.1149

Fiebelkorn, I. C., Saalmann, Y. B., & Kastner, S. (2013). Rhythmic sampling within and between objects despite sustained attention at a cued location. *Current Biology*, *23*(24), 2553–2558. doi:10.1016/j.cub.2013.10.063

Ho, H. T., Leung, J., Burr, D. C., Alais, D., & Morrone, M. C. (2017). Auditory sensitivity and decision criteria oscillate at different frequencies separately for the two ears. *Current Biology*, *27*(23), 3643–3649.e3. doi:10.1016/j.cub.2017.10.017

Hope, R. M. (2014). Rmisc: Ryan miscellaneous. *R package version*, *1.6.*

Huang, Q., & Luo, H. (2020). Saliency-based rhythmic coordination of perceptual predictions. *Journal of Cognitive Neuroscience*, *32*(2), 201–211. doi:10.1162/jocn_a_01371

Huang, Y., Chen, L., & Luo, H. (2015). Behavioral oscillation in priming: Competing perceptual predictions conveyed in alternating theta-band rhythms. *The Journal of Neuroscience*, *35*(6), 2830–2837. doi:10.1523/JNEUROSCI.4294-14.2015

Hurvich, C. M., & Tsai, C. L. (1989). Regression and time series model selection in small samples. *Biometrika*, *76*(2), 297–307. doi:10.1093/biomet/76.2.297

Lenth, R., Singmann, H., Love, J., Buerkner, P., & Herve, M. (2018). Emmeans: Estimated marginal means, aka least-squares means. *R package version*, *1*(1), 3.

Li, T., Wang, L., Huang, W., Zhen, Y., Zhong, C., Qu, Z., & Ding, Y. (2020). Onset time of inhibition of return is a promising index for assessing cognitive functions in older adults. *Journals of Gerontology: Psychological Sciences*, *75*(4), 753–761. doi:10.1093/geronb/gby070

Mazerolle, M. J. (2020). AICcmodavg: Model selection and multimodel inference based on (q)AIC(c). *R package version 2.3-1*, *2.3*(1).

Myung, J., Cavagnaro, D. R., & Pitt, M. A. (2016). Model evaluation and selection. *New handbook of mathematical psychology*, *1*, 552–598. doi:10.1017/9781139245913.011

Nichols, T. E., & Holmes, A. P. (2002). Nonparametric permutation tests for functional neuroimaging: A primer with examples. *Human Brain Mapping*, *15*(1), 1–25. doi:10.1002/hbm.1058

Padfield, D., & Matheson, G. (2018). Nls.multstart: Robust non-linear regression using AIC scores. *R package version*, *1*(0), 1–5.

Pavlov, Y. G., Adamian, N., Appelhoff, S., Arvaneh, M., Benwell, C. S. Y., Beste, C., . . . Mushtaq, F. (2021). #EEGManyLabs: Investigating the replicability of influential EEG experiments. *Cortex*, *144*, 213–229. doi:10.1016/j.cortex.2021.03.013

Raftery, A. E. (1995). Bayesian model selection in social research. *Sociological Methodology*, *25*, 111–163. doi:10.2307/271063

Rasch, B., Friese, M., Hofmann, W., & Naumann, E. (2014). G*power-ergänzungen. In *Quantitative methoden* (Vol. 2, pp. 65–91). Heidelberg: Springer.

Re, D., Inbar, M., Richter, C. G., & Landau, A. N. (2019). Feature-based attention samples stimuli rhythmically. *Current Biology*, *29*(4), 693–699.e4. doi:10.1016/j.cub.2019.01.010

Robinson, D., Hayes, A., & Couch, S. (2022). Broom: Convert statistical objects into tidy tibbles. *R package version 0.7.12*.

Schäfer, T., & Schwarz, M. A. (2019). The meaningfulness of effect sizes in psychological research: Differences between sub-disciplines and the impact of potential biases. *Frontiers in Psychology*, *10*(813), 1–13. doi:10.3389/fpsyg.2019.00813

Schwarz, G. (1978). Estimating the dimension of a model. *The Annals of Statistics*, *6*(2), 461–464. doi:10.1214/aos/1176348654

Singmann, H., Bolker, B., Westfall, J., Aust, F., & Ben-Shachar, M. S. (2022). Afex: Analysis of factorial experiments. *R package version*, *1.1*(0).

Song, K., Meng, M., Lin, C., Zhou, K., & Luo, H. (2014). Behavioral oscillations in attention: Rhythmic alpha pulses mediated through theta band. *The Journal of Neuroscience*, *34*(14), 4837–4844. doi:10.1523/JNEUROSCI.4856-13.2014

Su, Z., Wang, L., Kang, G., & Zhou, X. (2021). Reward makes the rhythmic sampling of spatial attention emerge earlier through alpha pulses. *Attention, Perception & Psychophysics*, 1–16. doi:10.3758/s13414-020-02226-5

Sugiura, N. (1978). Further analysts of the data by akaike' s information criterion and the finite corrections. *Communications in Statistics - Theory and Methods*, *7*(1), 13–26. doi:10.1080/03610927808827599

Thaler, L., Schütz, A. C., Goodale, M. A., & Gegenfurtner, K. R. (2013). What is the best fixation target? the effect of target shape on stability of fixational eye movements. *Vision Research*, *76*, 31–42. doi:10.1016/j.visres.2012.10.012

Wagenmakers, E. J. (2007). A practical solution to the pervasive problems of p values. *Psychonomic Bulletin & Review*, *14*(5), 779–804. doi:10.3758/BF03194105

Wagenmakers, E. J., & Farrell, S. (2004). AIC model selection using akaike weights. *Psychonomic Bulletin & Review*, *11*(1), 192–196. doi:10.3758/BF03206482

Wickham, H., Averick, M., Bryan, J., Chang, W., McGowan, L. D., François, R., … Yutani, H. (2019). Welcome to the tidyverse. *Journal of Open Source Software*, *4*(43), 1686. doi:10.21105/joss.01686
